# The Medical Society

**Published:** 1896-11-28

**Authors:** 


### Medical Progress and Hospital Clinics.

[*The Editor will be glad to receive offers of co-operation and contributions from members of the profession. All letters should be addressed to* The Editor, at the Office, 28 & 29, Southampton Street, Strand, London, W.C.]

### THE MEDICAL SOCIETY.

In a paper read last Monday Dr. Haig very tersely and definitely laid down his views as to the *rôle* played by uric acid in the production of various diseases. He maintained that, so far from uric acid, when swallowed by man, being converted into urea, it gets into the blood, and eventually appears as uric acid in the urine, grain for grain, in proportion to the quantity ingested; that in the normal metabolism of the body urea and uric acid are found in certain fixed proportions, viz., 35 of urea to one of uric acid, and that all the uric acid that exists or is excreted beyond this proportion is the result not of excessive formation, but of its introduction with the food; that the presence of uric acid beyond this proportion, or even in this proportion when the quantity of urea produced is large, causes a series of toxic symptoms which may be looked upon as uric acid diseases; but that these diseases are not signs of a uric acid diathesis leading to excessive production of uric acid, but are merely the result of taking in our food a substance, uric acid and zanthius, which can be easily eliminated from our diet without injury to nutrition. He believed, then, that when these matters were better understood, the uric acid diathesis, so called, would come to be regarded as a myth.

In the discussion various objections were raised to the absoluteness and inflexibility of the law of uric acid excretion as laid down by Dr. Haig, although the utility of his rules as a guide to treatment was generally acknowledged. The occurrence of uric acid in milk-fed infants was adduced as a proof that the acid was formed in the body. Dr. Vaughan Harley pointed to the excessive excretion in patients suffering from leucocythæmia, and stated that when a healthy person and one suffering from that disease were fed on precisely the same diet the leucocythæmic individual excreted nearly four times as much uric acid as the other. The same held good, he said, as to malignant disease, so much so that he would almost question a diagnosis of malignant disease of the liver if the uric acid were found not to be increased. Finally Dr. Freyer put the crushing conundrum: If uric acid comes from eating meat, why do such enormous numbers of the Hindu population of India suffer from calculus, seeing that they eat no meat at all?

Dr. Haig, in answer, thought that many experiments were invalidated by not being continued for a long enough time, as the body in certain conditions stored the uric acid, and it might be found in the spleen and other parts. As to the occurrence of calculus in India, he said it was most common in men and boys, and suggested that they got the best food which by general consent was the most nitrogenous. His answer to this and some other questions might, perhaps, have been strengthened if he had maintained, as he might with reason have done, that the formation of crystals or calculi of uric acid has to do with a change in the urine after it is secreted, and is but small proof of the existence of any excess of the acid either in the blood or in urine.

